# An Optimized, Chemically Regulated Gene Expression System for *Chlamydomonas*

**DOI:** 10.1371/journal.pone.0003200

**Published:** 2008-09-12

**Authors:** Paola Ferrante, Claudia Catalanotti, Giulia Bonente, Giovanni Giuliano

**Affiliations:** 1 Italian National Agency for New Technologies, Energy and the Environment (ENEA), Casaccia Research Center, Rome, Italy; 2 Dipartimento Scientifico e Tecnologico, University of Verona, Verona, Italy; Cairo University, Egypt

## Abstract

**Background:**

*Chlamydomonas reinhardtii* is a model system for algal and cell biology and is used for biotechnological applications, such as molecular farming or biological hydrogen production. The *Chlamydomonas* metal-responsive *CYC6* promoter is repressed by copper and induced by nickel ions. However, induction by nickel is weak in some strains, poorly reversible by chelating agents like EDTA, and causes, at high concentrations, toxicity side effects on *Chlamydomonas* growth. Removal of these bottlenecks will encourage the wide use of this promoter as a chemically regulated gene expression system.

**Methodology:**

Using a codon-optimized *Renilla* luciferase as a reporter gene, we explored several strategies to improve the strength and reversibility of *CYC6* promoter induction. Use of the first intron of the *RBCS2* gene or of a modified TAP medium increases the strength of *CYC6* induction up to 20-fold. In the modified medium, induction is also obtained after addition of specific copper chelators, like TETA. At low concentrations (up to 10 µM) TETA is a more efficient inducer than Ni, which becomes a very efficient inducer at higher concentrations (50 µM). Neither TETA nor Ni show toxicity effects at the concentrations used. Unlike induction by Ni, induction by TETA is completely reversible by micromolar copper concentrations, thus resulting in a transient “wave” in luciferase activity, which can be repeated in subsequent growth cycles.

**Conclusions:**

We have worked out a chemically regulated gene expression system that can be finely tuned to produce temporally controlled “waves” in gene expression. The use of cassettes containing the *CYC6* promoter, and of modified growth media, is a reliable and economically sustainable system for the temporally controlled expression of foreign genes in *Chlamydomonas*.

## Introduction

*Chlamydomonas reinhardtii* is a model system for the biology of green algae. The recent completion of its genome sequence [1] has paved the way for a post-genomics effort, aimed at understanding the function of the majority of *Chlamydomonas* genes. Stable chloroplast and nuclear transformants can be obtained easily, and a large number of mutants and genetic resources are already available (www.chlamy.org). Scaling up of cultures to large volumes is rapid and biomass production is cost-effective, making *Chlamydomonas* an interesting system for the production of heterologous proteins [2] and of biohydrogen [3].

Gene cassettes allowing chemically regulated gene expression are an essential part of a post-genomics toolkit. Several such systems have been described in plants [4]. These systems allow the temporal or developmental control of the expression of specific genes, thus facilitating the precise determination of their function. They also allow the precise control of the expression of potentially toxic gene products, for industrial or pharmaceutical uses. Ideally, a chemically inducible gene expression system should have the following characteristics:

- the inducer should be active at very low (micromolar) concentrations
- the induction should be reversible by the addition of micromolar concentrations of an antagonist of the inducer
- basal expression levels should be very low, while induced expression levels should be very high
- the expression should respond quantitatively and rapidly to the levels of inducer and its antagonist
- addition of the inducer, followed by the antagonist should allow transient gene expression, useful for the expression of a gene product at precise moments during the growth cycle of an organism
- both the inducer and its antagonist should be non-toxic

In *C. reinhardtii,* expression of heterologous proteins presents several difficulties. The first problem is represented by the unusual codon bias of the nuclear genes that is highly G-C rich (62%), so that codon optimization must be performed on any gene for which high levels of protein expression are desired [5], [6]. Additionally, expression levels of optimized foreign genes may vary considerably due to position effects or silencing mechanisms [7]. Another feature of most *Chlamydomonas* nuclear genes is the presence of several small introns in their coding sequences that exert a positive role in gene expression. In particular, the first intron from the gene that encodes the small subunit of ribulose bisphosphate carboxylase (*RBCS2*) was found to act as an enhancer-like element. This intron (*Rb-int*) can increase levels of the *ble* selection marker up to 30-fold [8].

Constitutive promoters commonly used for *Chlamydomonas* transformation are the *RBCS2* promoter [9], the *HSP70A-RBCS2* tandem promoter [10] and the *PSAD* promoter [11], whereas inducible promoters are *NIT1*, *CA1* and *CYC6*. The *NIT1* promoter is induced by ammonium starvation [12], the *CA1* promoter by low CO_2_ [13], whereas the *CYC6* promoter is induced by copper (Cu) depletion or nickel (Ni) or cobalt addition [14]. The advantages of using inducible instead of constitutive promoters are that potentially toxic gene products can be expressed only after reaching high cell densities, thus optimizing protein yield, and that strategies can be pursued for the conditional silencing of essential genes. However, none of the above-mentioned promoters has been developed into a robust, widely used chemically regulated gene expression cassette.

The *Chlamydomonas CYC6* gene encodes cytochrome c_6_, that replaces, in Cu-limiting conditions, the Cu-containing protein plastocyanin in photosynthetic electron transfer. Plastocyanin is the major copper protein of *Chlamydomonas*, acting as biological sink of Cu. When Cu is limiting, plastocyanin is degraded to facilitate its redistribution to other more physiologically important copper enzymes such as cytochrome oxidase. Cu deficiency switches on the *CYC6* promoter, while addition of sub-micromolar concentrations of Cu ions turns it off [15]. The *CYC6* promoter is also activated by Ni and cobalt [14] and oxygen deficiency [16]. Another possible way of induction, i.e. depletion of Cu by specific chelators, has been suggested [17] but not shown to work.

Recently, the *CYC6* promoter has been used to construct an elegant gene switch for chloroplast genes [18]: a *CYC6:NAC2* nuclear transgene, introduced in a *nac2* background, allows Cu-repressible expression of the plastid *PsbD* gene (or of any plastid transgene cloned downstream of the *PSBD* 5′-UTR). Following *PSBD* repression by Cu, the culture becomes rapidly anaerobic and a burst of hydrogen production is observed [18]. This burst is, however, only transient, as the *CYC6* promoter is rapidly re-induced as soon as anaerobic conditions are established. The availability of methods able to strongly and reversibly induce the *CYC6* promoter would extend the range of applications of this gene switch.

In the present work we explore several strategies to improve *CYC6* activation by Ni and Cu-specific chelators. The use of the first intron of the *RBCS2* gene in a specific orientation and position respect to the *CYC6* promoter results in an increase of *CYC6* activity upon Ni and TETA supplement in the TAP modified media. Induction levels of the *CYC6* promoter increase significantly in TAP media with modified transition metal content. The use of the Cu-specific chelators, such as TETA, is proven to be a viable alternative strategy to induce the *CYC6* promoter. TETA activation is readily reversible upon Cu addition, allowing, for the first time in an algal, plant or mammalian system, the production of transient “waves” in gene expression.

## Results and Discussion

### Metal inducibility of a *CYC6:rLuc* construct

In order to provide a sensitive and reliable gene reporter system, we used a synthetic gene encoding *Renilla reniformis* luciferase (*cRLuc*) [6], whose sequence has been adapted to the average codon usage of nuclear genes from *C. reinhardtii*. The estimated half-life of the cRLuc protein in the *Chlamydomonas* cytoplasm is <2 hours [6]. This is particularly important for studying rapid fluctuations in promoter activity.

**Figure 1:**
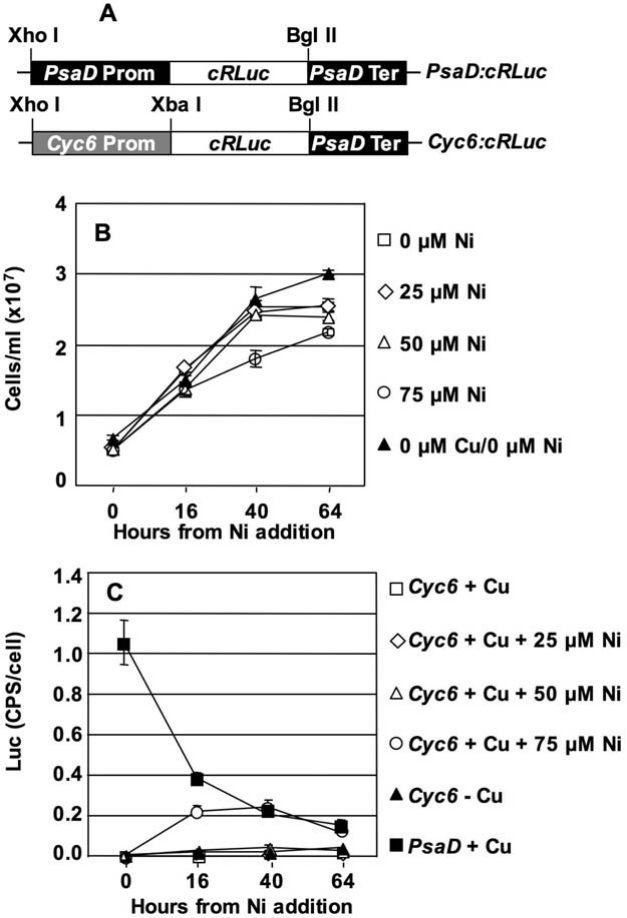
Comparative expression of *PSAD* and *CYC6* promoters in TAP medium. A: Schematic maps of the *PSAD:cRLuc* and *CYC6:cRLuc* constructs. B: Growth curves in Cu-deficient and Cu-replete medium, and in the presence of different Ni concentrations. C: LUC activity driven by *PSAD* and *CYC6* in Cu-deficient and Cu-replete medium, and in the presence of different Ni concentrations.

*cRLuc* was cloned downstream of the strong constitutive *PSAD* promoter [11] and of the *CYC6* promoter [14], [15] (Figure 1A) and transformed into *Chlamydomonas*. 24 *PSAD:cRLuc* and 24 *CYC6:*c*RLuc* transformants were selected in TAP medium containing paromomycin, and then tested for LUC activity on TAP medium, with or without 75 µM Ni (data not shown). Figure 1C shows LUC activity curves of the highest-expressing *PSAD* transformant in normal TAP medium and of the highest-expressing *CYC6* transformant in normal (Cu-replete) and Cu-deficient TAP medium. The *CYC6* promoter was also induced by adding different Ni concentrations, from 25 to 75 µM, to Cu-replete medium. The *PSAD* promoter shows, in early log phase, a high activity, which decreases over time. To the opposite, the *CYC6* promoter has a weak activity in Cu-deficient medium (0.3× *PSAD* activity 40 hours after Ni addition, Figure 1C), and no activity in Cu-replete medium. The use of acid-treated glassware and plasticware to remove Cu ion traces [17] improved only marginally *CYC6* expression in Cu-deficient medium (data not shown). In Cu-replete medium, the addition of Ni at 25 µM does not induce the *CYC6* promoter at all while Ni at 50 µM results in a weak induction (0.2× *PSAD*). A higher induction is reached with Ni at 75 µM (1.2× *PSAD*) but toxicity effects like inhibition of cell growth (Figure 1B) and a moderate degree of chlorosis (data not shown) start also to be evident.

Using Northern blots, Quinn *et al*. [14] observed activation of *CYC6* transcription by 25 µM Ni. The absence of induction of LUC activity by 25 µM Ni observed here may be due to strain-dependent variation, or to the different sensitivity of Northern blots and LUC assays. Whatever the case, the comparison of the LUC activity levels driven by *PSAD* and of those driven by *CYC6* indicates clearly that the latter is a rather weak promoter in TAP medium, when induced with non-toxic concentrations of Ni.

### Increasing *CYC6* promoter strength by optimization of medium composition

Transition metals are added to the TAP medium as EDTA-complexes in a solution known as Hutner trace solution [19]. This solution was originally developed for the growth of bacteria and up to now has not been optimized for the metabolism of *Chlamydomonas* strains [20]. Although transition metals are essential for *Chlamydomonas* growth, the minimum required concentrations are presumably much lower than those provided by the Hutner solution. Table 1 shows the concentration of each transition metal provided by the Hutner solution and the minimal estimated concentration required to support *Chlamydomonas* growth up to the stationary phase [20]. It is clear from Table 1 that all transition metals are present in large excess. Some of the transition metals are likely to interfere with Cu signal transduction, like cobalt ions that promote *CYC6* activation even in fully Cu-replete cells [14]. In addition, an excess of Cu is likely to antagonize *CYC6* induction by nickel. Taking these facts into account, two new trace solutions were prepared (Table 1). ENEA1 solution is identical to the Hutner solution except for Cu, which is 0.3 µM instead of 6 µM (final concentration). ENEA2 contains 0.3 µM Cu and lowered concentrations of all other transition metals and the chelator EDTA. The rationale of lowering EDTA concentration is to avoid having an excess of EDTA, that may chelate nickel with high affinity (the stability constant of EDTA-Ni is 18.56 [21]), thus antagonizing *CYC6* activation by this metal.

**Table 1:** Trace element concentrations (µM).

	Minimum Required*	TAP	TAP ENEA1	TAP ENEA2
Zn	1.7	77	77	3
Mn	1.7	26	26	3
Fe	3.3	18	18	5
Co	0.003	7	7	0.1
Cu	0.3	6	0.3	0.3
Mo	0.003	1	1	0.1
EDTA	-	134	134	15

* Calculated from data in [20].

**Figure 2:**
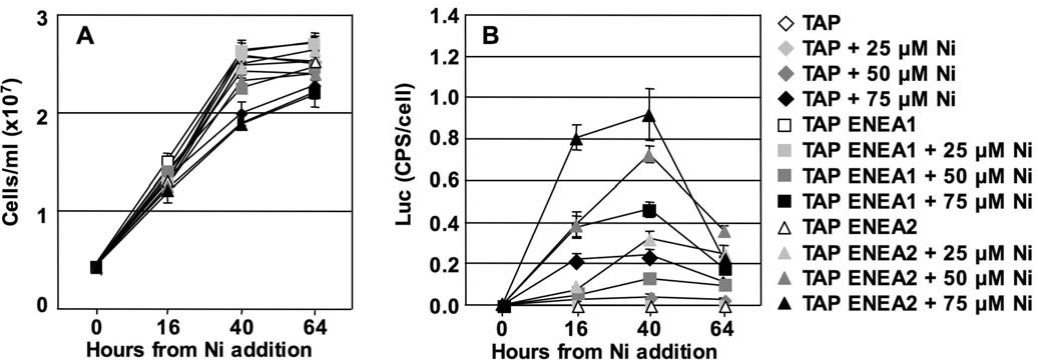
Effect of modified TAP media on *Chlamydomonas* growth and on *CYC6* promoter expression. A: Growth curves in media with different transition metal/EDTA composition, in the presence of different Ni concentrations. For composition of the different media, see Table 1. B: LUC activity in the above media at different Ni concentrations.

Figure 2 shows the growth and LUC activity curves of the *CYC6:cRLuc* transformant grown in canonical TAP medium, or with ENEA1 or ENEA2 trace element compositions, and induced with 25 µM, 50 µM, and 75 µM Ni. The growth curves of the non induced cultures show that the ENEA trace solutions provide concentrations of transition metals sufficient to sustain *Chlamydomonas* growth up to the stationary phase while Ni at 75 µM causes cell growth inhibition irrespective of media tested (panel A).

LUC activity curves (panel B) show that 25 µM Ni is effective in activating the *CYC6* promoter only in TAP ENEA2 medium, whereas no induction is observed in TAP and TAP ENEA1 media. At higher Ni concentrations (50 and 75 µM), Ni induces the *CYC6* promoter in all three media tested, but maximum *CYC6* induction differs significantly among TAP media. At 50 µM Ni, the *CYC6* activity at 40 hours, is 0.2× *PSAD* in TAP medium, 0.6× *PSAD* in TAP ENEA1 and 3.5× *PSAD* in TAP ENEA2 medium (Table 2). This means a 17.5-fold improvement in expression between classical TAP and TAP ENEA2, without detectable toxicity effects.

**Table 2:** Strength of the *CYC6* promoter relative to *PSAD* in different growth media*.

	TAP	TAP ENEA1	TAP ENEA2
25 µM Ni	<0.01	<0.01	1.60
50 µM Ni	0.20	0.60	3.50
75 µM Ni	1.20	2.20	4.40

* Promoter strength has been calculated 40 hours after Ni addition (70 hours after subculture), when *CYC6*, but not *PSAD*, expression is maximal. Therefore, the data reflect *CYC6* relative expression in different growth media, rather than absolute *CYC6/PSAD* ratios at the relative expression peaks.

These findings suggest that both the levels of Cu and the levels of transition metals and EDTA regulate *CYC6* induction by Ni. The increased induction levels observed in TAP ENEA1 *versus* classical TAP medium can be explained considering that copper antagonizes induction by Ni, since the difference between these two media is only the copper content. On the other hand, the increased levels of *CYC6* induction in TAP ENEA2 medium compared to TAP ENEA1 medium can be explained by an increased Ni uptake by *Chlamydomonas* cells. Transport of transition metal cations like Zn, Cd, Co, Ni or Mn across the plasma membrane is mediated by non-specific cation transporters [22] and hence, in TAP ENEA2 medium, where all of the cations are reduced, Ni uptake is probably enhanced.

### Reversibility of the *CYC6* promoter induction: Ni/EDTA system

An ideal inducible system should be readily reversible by the addition of an antagonist of the inducer, acting at micromolar concentrations. Using Northern blotting, Quinn *et al.* [14] have shown that EDTA, added 5 hs after Ni addition, is able to prevent induction of *CYC6* transcript levels by Ni. Since, at this time, the *CYC6* transcript is still undetectable, this cannot formally be considered as a reversion, but rather as a lack of induction.

**Figure 3:**
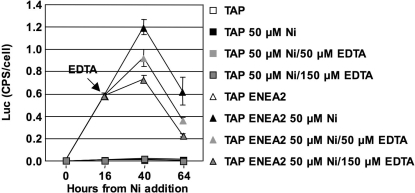
Induction by Ni is not reversible by EDTA. LUC activity, induced with 50 µM Ni in TAP and TAP ENEA2 medium, and supplemented with different concentrations of EDTA 16 hours after Ni addition.

To verify if the Ni/EDTA system can be used for driving the reversible expression of a heterologous protein, we induced the promoter with 50 µM Ni in TAP ENEA2 and canonical TAP medium. EDTA was added at the final concentration of 50 and 150 µM (Figure 3) 16 hs after Ni addition, when LUC activity becomes detectable.

As can be seen (Figure 3), in both media induction of the *CYC6* promoter is poorly reversible by EDTA, even when added at very high concentrations. In TAP ENEA2, LUC activity keeps increasing after EDTA addition, even if at a lower pace. In all cases, LUC activity is well above background levels even 48 hours after EDTA addition. Similar lack of reversion is observed after induction with 25 µM Ni in TAP ENEA2 medium or with 50 µM Ni in canonical TAP medium (Figure S1).

A different option to reversibly regulate the *CYC6* promoter would be to add Cu to cultures induced by Ni. We tried adding 25 µM and 100 µM Cu concentrations 16 hours after induction by Ni (25, 50 and 75 µM) in canonical TAP and in TAP ENEA2 media and we found them ineffective in switching off the *CYC6* promoter (Figure S2). These results are in agreement with the model proposed by Kropat *et al.* [23], in which Ni binds at the Cu-sensing site of the Cu Response Regulator 1 (CRR1) with high affinity, precluding subsequent displacement by Cu. The irreversible nature of Ni binding to the CRR1 regulator is also suggested by the fact that EDTA, added 40 hs after Ni addition is completely ineffective in switching off the *CYC6* promoter (Figure S3).

These data, taken together, seem to indicate that the rate limiting step for achieving high level of *CYC6* induction is the amount of Ni inside the cell available to bind the CRR1 regulator. Our results indicate that high levels of *CYC6* induction can be obtained in transition metal-poor media, where Ni uptake is probably increased compared to standard media. The addition of EDTA at early times after Ni induction prevents efficient Ni uptake and *CYC6* induction, while later addition is not able to revert the action exerted by the Ni that has been already taken up. From these results we conclude that the Ni/EDTA system is not ideal in driving reversible expression of a heterologous protein put under the control of the *CYC6* promoter.

### *CYC6* induction by specific Cu chelators

Since the *CYC6* promoter is naturally responsive to Cu deficiency in the growth medium, Cu-specific chelators may provide an alternative strategy for inducing gene expression. We tested several chelators, both with a broad chelating activity and specific to Cu (listed in Table 3). Several of the Cu-specific chelators are used as remedies in Wilson's disease, a genetic disorder in which copper accumulates in the brain and the liver, causing neuropsychiatric symptoms and liver disease [24].

**Table 3:** Characteristics of the different Cu chelators tested.

Short name	Full name	Comments	Reference
PHE	1,10-phenanthroline	Cu-chelator	[32]
CDTA	trans-1,2-diaminocyclohexane-N,N,N′,N′-tetraacetic acid	Cu-chelator. Remedy in metal poisoning	[33]
TETA	triethylentetramine	Cu-chelator. Remedy in Wilson's disease	[34]
DDC	sodium diethyldithiocarbamate	Cu-chelator	[32]
DPA	D-penicillamine	Cu-chelator. Remedy in Wilson's disease	[34]
BCS	bathocuproinedisulfonic acid	Cu-chelator	[35]
IM	1,3,5-*cis*,*cis,-*-triaminocyclohexane-N,N′,N″-tris-(2-methyl-(N-methylimidazole))	Cu-chelator	[25]
DOTA	1,4,7,10-Tetraazacyclododecane-1,4,7,10-tetraacetic acid	Cu-chelator	[36]

The chelators were tested in TAP ENEA2 medium, since in this medium transition metals and EDTA are low, providing a low buffering capacity against the addition of chelators. In theory, the concentration of chelator causing preferential chelation of Cu over other essential cations can be calculated from the stability constants of the chelator-metal complexes. In practice, *in vivo* chelation reactions may differ extensively from what would be expected on the basis of the chemical knowledge about the metal and the chelating agent. Therefore, in this initial screening we used 2 µM and 10 µM concentrations of all chelators.

**Figure 4:**
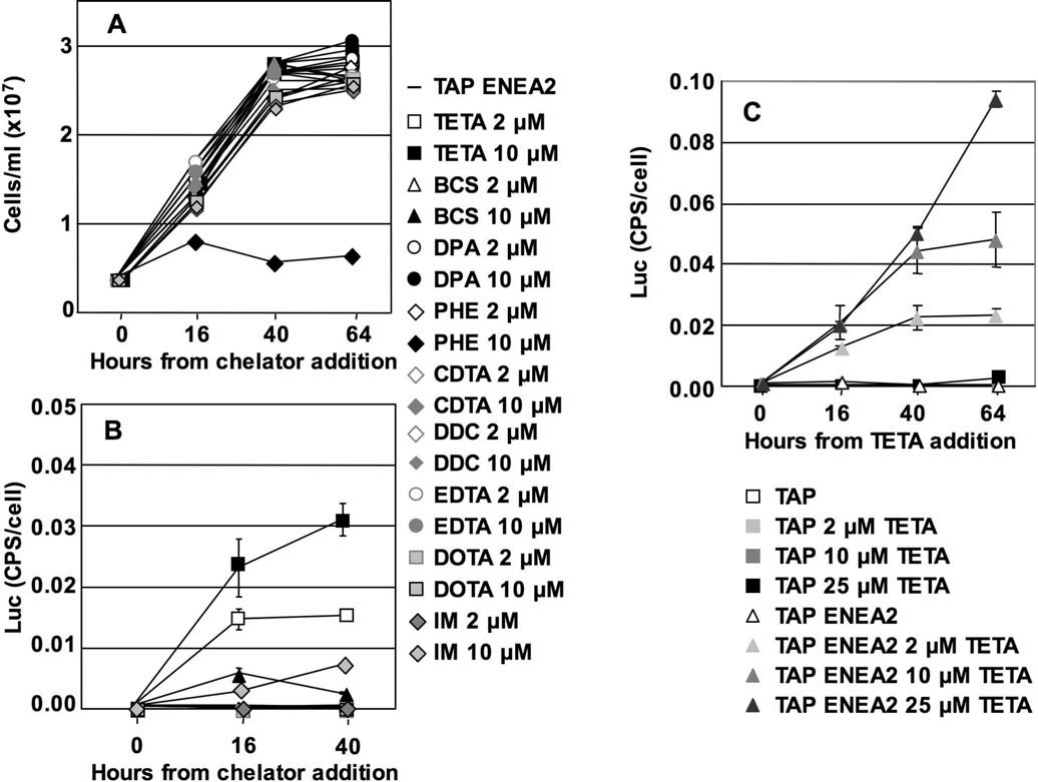
Induction of *CYC6* promoter expression by different chelators in modified TAP media. A: Growth curves in TAP ENEA2 medium in the presence of different chelators. The chelator full names and references are given in Table 3. B: LUC activity in the above conditions. C: Induction of LUC activity by TETA in classical and modified TAP media.

The only chelator showing toxicity effects (at 10 µM) is 1,10-phenanthroline, whereas all the other chelators do not have negative effects on *Chlamydomonas* growth, in spite of the low transition metal content of TAP ENEA2 medium (Figure 4A). Several chelators, like TETA (2 and 10 µM), BCS (10 µM) and IM (10 µM) result in a measurable activation of the *CYC6* promoter (Figure 4B).

We tested a broader range of TETA in TAP and TAP ENEA2 media (Figure 4C). As expected, activation by TETA is much more efficient in TAP ENEA2 than in classical TAP. Activation by TETA in classical TAP medium is observed only at 25 µM and is very weak (0.006× *PSAD*) whereas 2, 10 and 25 µM are effective in activating the *CYC6* promoter in TAP ENEA2 medium. These results are explained considering that TETA is a copper-specific chelator and that classical TAP medium has a large amount of copper that cannot be completely sequestered by TETA. Figure 4C also shows that the *CYC6* promoter responds quantitatively to the levels of TETA added (from 2 µM to 25 µM in TAP ENEA2 medium) reaching, after 64 hours, induction levels from 0.2× *PSAD* at 2 µM TETA to 0.6× *PSAD* at 25 µM TETA. Growth curves (not shown) indicate that TETA is not toxic at all the concentrations tested. In this way the expression of a protein can be quantitatively modulated by the levels of TETA without interfering with cell growth. It must be noted that, although induction by Ni reaches higher activity levels, at low concentrations (10 µM) TETA is a more potent inducer than Ni (Figure S3).

### Reversibility of the *CYC6* promoter induction: TETA/Cu system

**Figure 5:**
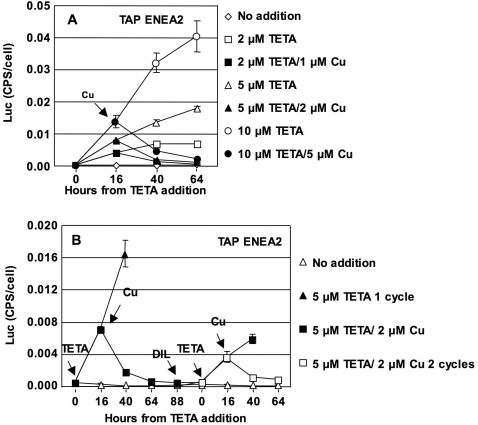
Induction by TETA is reversible by Cu. A: LUC activity in cultures induced with TETA, and supplemented with different concentrations of Cu 16 hours later. B: LUC activity in cultures induced with 5 µM TETA and repressed with 2 µM Cu for two subsequent cycles.

At all the TETA concentrations tested, LUC activity is already detectable at 16 hours and increases steadily until 64 hours. When Cu is added at 16 hours (1, 2 and 5 µM respectively for 2, 5 and 10 µM TETA), LUC activity drops, at 64 hours, to the levels of the non induced samples (Figure 5A). This creates a reversible wave-like pattern, in which gene expression is switched on by the addition of TETA, and then off again by the addition of Cu. Even if added at 40 hs after TETA induction, Cu reverses readily *CYC6* activation (Figure S3).

In principle, the TETA/Cu reversible system should be usable for more than one cycle of activation-repression. In order to verify this hypothesis, gene expression was induced with 5 µM TETA and repressed with 2 µM Cu, added 16 hours later (Fig. 5B). After 88 hours, the cultures have reached stationary phase and are diluted 1∶20 with fresh TAP ENEA2 medium. In this new growth cycle, TETA is again added at 30 hours and Cu 16 hours later. In both cases, a second cycle of activation-repression is obtained. The maximum levels of *CYC6* induction are lower in the second cycle with respect to the first one, probably due to carry-over of the Cu used for repression during the dilution of the medium. Similar trends are observed after induction with 2 µM TETA, followed by repression by 1 µM Cu (Figure S4)

### Increasing *CYC6* promoter strength with the *RBCS2* intron

Studies of gene expression in *C. reinhardtii* have shown that introns play an important role in regulating gene expression levels, altering nuclear export, stability of transcripts or the rate of transcription. In particular, the first intron of *RBCS2* gene (*Rb-int*) has been widely used as a transcriptional enhancer, able to increase expression up to 30-fold, regardless of its orientation or position relative to the *RBCS2* promoter [8].

**Figure 6:**
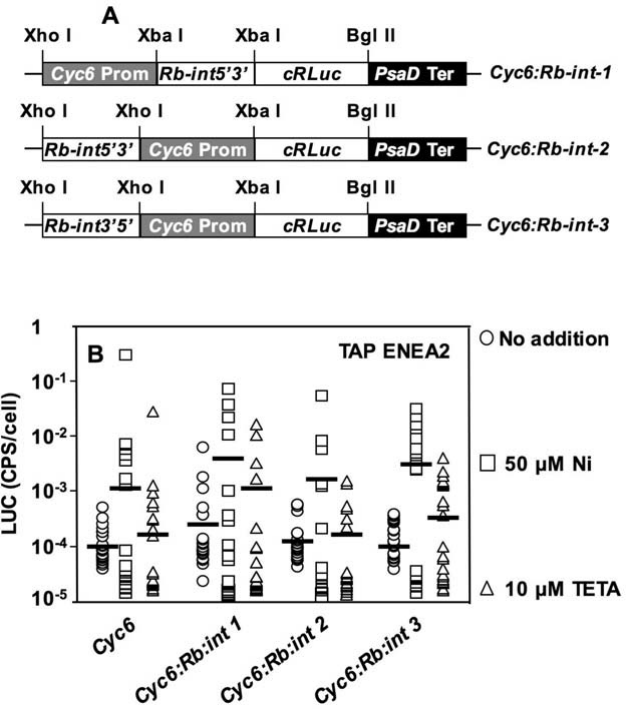
Effect of the first intron of *RBCS2* on *CYC6* promoter expression. A: Schematic maps of the *CYC6:Rb-int1*, *2* and *3* constructs. B: LUC activity, 40 hours after inducer addition, of 24 independent transformants for each of the constructs shown in panel A and for the *CYC6* construct. LUC activity values are in log scale. Horizontal bars represent the average expression of the 24 transformants.

As an alternative strategy to increase the strength of the *CYC6* promoter, *Rb-int* was cloned upstream (in both orientations) and downstream of said promoter (Figure 6A). Luciferase activity was determined, in TAP ENEA2 medium, uninduced or after induction with 50 µM Ni or 10 µM TETA, on 24 transformants for each of the five constructs shown in figures 1A and 6A. The results (Figure 6B) show that the *Rb-int1* construct has the highest expression levels after Ni/TETA addition. However the basal level of activity in the absence of Ni/TETA is high (Figure 6B), indicating that *Rb-int* acts as a constitutive enhancer downstream the *CYC6* promoter. *Rb-int2* transformants behave as *CYC6* transformants (Figure 6B). To the opposite, the induction of *Rb-int3* transformants by Ni and TETA is higher than that of *CYC6* transformants (Figure 6B). From these results we conclude that *Rb-int* behaves as a constitutive enhancer downstream of *CYC6* whereas it exerts a positive effect on *CYC6* induction by Ni and TETA when placed in 3′-5′ orientation, upstream of the *CYC6* promoter.

### Conclusions

We have shown that, after optimization of the *Chlamydomonas* growth media, expression of the *CYC6* promoter is strongly induced by non-toxic concentrations of Ni, or, to lower levels, of a specific Cu chelator, such as TETA. The activity induced by Ni is increased up to 18-fold in a transition metal and EDTA - poor medium, TAP ENEA2, with respect to classical TAP.

Ni induction is poorly reversible by EDTA. EDTA acts probably by preventing the penetration of Ni into the cell and, if added at early stages after induction [14]it blocks *CYC6* activation. On the contrary, if it is added at later stages of induction, EDTA is unable to completely revert Ni induction.

In TAP ENEA2 medium, the *CYC6* promoter is activated, at low levels, by the addition of a specific Cu-chelator, such as TETA. TETA is not toxic at all the concentrations tested and its induction is readily and completely reversible by Cu, this resulting in a transient “wave” in gene expression. Several Cu chelators, developed for clinical applications [25] are now being tested, and some of them are more effective *CYC6* inducers than TETA, while retaining reversibility by Cu.

Finally, the use of the first intron of the *RBCS2* gene in a specific orientation and position with respect to the *CYC6* promoter results in an increased inducibility of *CYC6* by Ni and TETA.

To our knowledge, this is the first time that a temporally reversible tuning of gene expression (turn on by an inducer, followed by turn off by an antagonist) is described in a chemically regulated system for algal, plant, or mammalian cells. The Tet-on, Tet-off system used in mammalian cells [26], functions in a fundamentally different way: in that case, the same chemical can act as an inducer or a repressor, depending on the construct used. A Dex-on, Tet-off system has been described in plants [27], but in that case the administration of the inducer and the antagonist are simultaneous, and do not give rise to gene expression “waves” like the ones described here.

These findings open new perspectives for basic biology applications as well as for biotechnological applications. *Chlamydomonas* has been proposed as a “cell factory” system for the production of heterologous proteins [28]. Some of these proteins may be toxic to the cell, and thus require an inducible, rather than a constitutive expression system. The system proposed here uses simple media, with very low concentrations of transition metals and EDTA, which might interfere with protein activity/purification. Induction can be achieved also in the absence of Ni, whose presence might interfere with some applications, such as the use of His affinity tags. Preparation of the medium does not require laborious acid washes of the glassware and plasticware, or use of ultra pure reagents, as in the case of induction by Cu depletion [17]. Finally, the system described here can be used to drive Ni- and TETA-inducible chloroplast gene expression [18]. *Chlamydomonas* is also used for the biotechnological production of hydrogen through indirect photobiolysis [3]. In the currently used system, downregulation of Photosystem II activity, leading to hydrogen production, is triggered by cycling *Chlamydomonas* cultures between sulphur-replete and sulphur-depleted medium. The method presents evident challenges, such as the difficulty of centrifuging the huge volumes of algal cultures needed for making hydrogen production economically interesting. The use of the TETA/Cu reversible system described here could be used to trigger several subsequent cycles of gene expression/silencing in a cheap, energy-efficient way.

## Materials and Methods

### Strains and culture conditions

The cell wall-deficient *Chlamydomonas reinhardtii* strain *cw15* [29] was used for all experiments. Cells were grown photomixotrophically in TAP medium [29], supplemented with 1% (w/v) sorbitol at 25°C under irradiation (16 L:8 D) with fluorescent white light (40 µE m^−2^ s^−1^). TAP medium was prepared using standard purity chemicals and MilliQ-purified water. All the glassware and plasticware was rinsed three times with MilliQ-purified water. ENEA1 and ENEA2 trace solutions were prepared according to [29] with modifications as in Table 1. TAP ENEA2 medium was supplemented with NaCl (200 µM final concentration). Nuclear transformation was performed as described [30]. Transformants were selected on paromomycine (10 µg/ml)–containing TAP agar plates and recovered 10 days after plating. For the experiment shown in Figure 6B, colonies were inoculated in duplicate in 250 µl of TAP ENEA2 medium in 96-well plates (Greiner bio-one, catalog number 655180), grown to the stationary phase at 600 rpm on a rotary shaker and then diluted 1∶20. 30 hours after the dilution, Ni and TETA were added at 50 and 10 µM, respectively. For all other experiments, stationary phase cultures of high-expressing *CYC6* and *PSAD* transformants were diluted 1∶20 in 2.5 ml of media in 24-well blocks (Qiagen, cat. 19583) and grown at 160 rpm on a rotary shaker. The plates were covered with Breathe-Easy membrane (Diversified Biotech, cat. BEM-1) to prevent evaporation without limiting gaseous and light exchange. Inducers (Ni, TETA) were added 30 hours after inoculum, when cell density had reached approx. 5×10^6^ cells/ml. Volumes were monitored throughout the growth curve, and evaporation was always <5% of the total volume. For the experiment shown in Figure 5B, the cultures from the first cycle were diluted 1∶20 88 hours after TETA addition. After 30 hours, TETA was added and, 16 hours later, Cu was added.

### Plasmid construction

Plasmid pSL18 [31], carrying an expression cassette composed of the *PSAD* promoter and polyadenylation site and a paromomycin resistance cassette, was used for all subsequent manipulations. The 874-bp XhoI-XbaI fragment containing the *PSAD* promoter was excised and substituted with a 931-bp fragment corresponding to the *CYC6* promoter+5′UTR (−852 to +79 with respect to the start site of transcription) [15]; this fragment was amplified by PCR from genomic DNA with primers adding XhoI and XbaI sites to 5′ and 3′ ends respectively and cloned in pSL18. The unique NotI restriction site in pSL18 was disrupted by filling of 3′ recessed ends after digestion. Then a polylinker sequence (50 bp long) containing the unique restriction sites StuI, NotI, PstI, SpeI, BglII, EcoRI, FseI was cloned in the XbaI site by annealing two primers with the following sequence:

Primer forward:

CTAGAGGCCTGCGGCCGCCTGCAGACTAGTAGATCTGAATTCGGCCGGCC

Primer reverse:

CTAGGGCCGGCCGAATTCAGATCTACTAGTCTGC AGGCGGCCGCAGGCCT

The synthetic gene encoding the *Renilla reniformis* luciferase (cRLuc) was cloned downstream of the *CYC6* promoter in the XbaI and BglII sites, forming plasmid pSL18:CYC6:cRLuc. Then, the XhoI-XbaI fragment containing the *CYC6* promoter was replaced with a 874-bp XhoI-XbaI fragment containing the *PSAD* promoter obtained through digestion of pSL18. Since the vector sequences downstream of the *PSAD* promoter (60 bp) contain a NdeI site (containing an ATG codon in frame with the *cRluc* gene), they were removed by digestion with NdeI and XbaI, filling with Klenow polymerase, and religation. The vector obtained was named pSL18:PSAD:cRLuc.

The constructs containing the first intron of *RBCS2* (*Rb-int*) upstream and downstream of the *CYC6* promoter were obtained by cloning the corresponding sequences in the XhoI (upstream) and XbaI (downstream) sites. *Rb-int* was isolated from the pSL18 plasmid by PCR.

### Ni- and chelator-induced gene expression

Cultures were supplemented with NiCl_2_, CuCl_2,_ and chelators (Table 3) from 1000× stock solutions. All chelators were ACS-grade and were purchased from Sigma-Aldrich with the exception of DOTA and IM that were kindly donated by M.W. Brechbiel. Growth was routinely monitored by reading the A_595_ with a Victor3 1420 Microplate Reader (Perkin-Elmer), and the number of cells was deduced using a conversion factor deduced by counting cultures at different densities with a haemocytometer. 100 µl of cell suspension was collected at the times indicated and centrifuged at 2,600×g for 20 minutes at 4°C in a 96-well PCR plate. Cell pellets were frozen in liquid nitrogen and stored at −80°C until used.

### Luciferase assay

Luciferase assay was performed using the *Renilla* Luciferase Assay System (Promega, cat. E2820) according to the manufacturer's instructions. Frozen cell pellets in multiwell plates were resuspended in 40 µl of 1× lysis buffer, lysed at room temperature for 15 minutes on a rotary shaker (750 rpm) and then incubated on ice until assayed. In these conditions, LUC activity remains stable for at least 1 hour (data not shown). Since LUC activity decays during the assay, with a half-life of approx 3 minutes, the assay was performed on 8 samples at a time operating with a multichannel pipet. 5 µl of each lysate was added to 25 µl of assay buffer in Optiplate 384-well plates (Perkin-Elmer cat. 6007290), supplemented with 1× coelenterazine, at room temperature, mixed for 3 seconds and luminescence was recorded for 2 seconds using a Victor3 1420 Microplate Reader (Perkin-Elmer). For each experimental point, two biological replicas (separate cultures) and two technical replicas (separate assays) were assayed, for a total of four replicas.

## Supporting Information

Figure S1 (0.05 MB PDF)

Figure S2 (0.05 MB PDF)

Figure S3 (0.05 MB PDF)

Figure S4 (0.05 MB PDF)
