# Supplementary material for: An Optimized, Chemically Regulated Gene Expression System for Chlamydomonas
Source: PLoS One. 2008 Sep 12;3(9):e3200. doi: 10.1371/journal.pone.0003200 (PMC2527658; doi:10.1371/journal.pone.0003200)
Supplement: Figure S1 — (0.05 MB PDF) [file pone.0003200.s001.pdf]

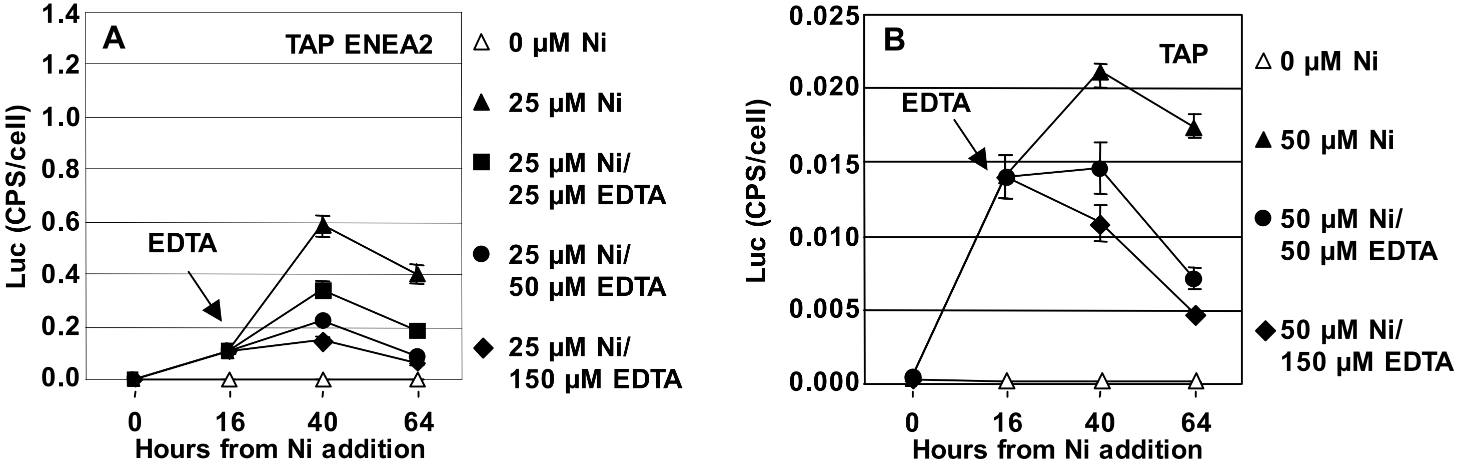

Figure S1: LUC activity in cultures induced with 25 µM Ni in TAP ENEA2 medium (A), or with 50 µM Ni in TAP medium (B), and supplemented with different concentrations of EDTA 16 hours after Ni addition.
