# Supplementary material for: An Optimized, Chemically Regulated Gene Expression System for Chlamydomonas
Source: PLoS One. 2008 Sep 12;3(9):e3200. doi: 10.1371/journal.pone.0003200 (PMC2527658; doi:10.1371/journal.pone.0003200)
Supplement: Figure S2 — (0.05 MB PDF) [file pone.0003200.s002.pdf]

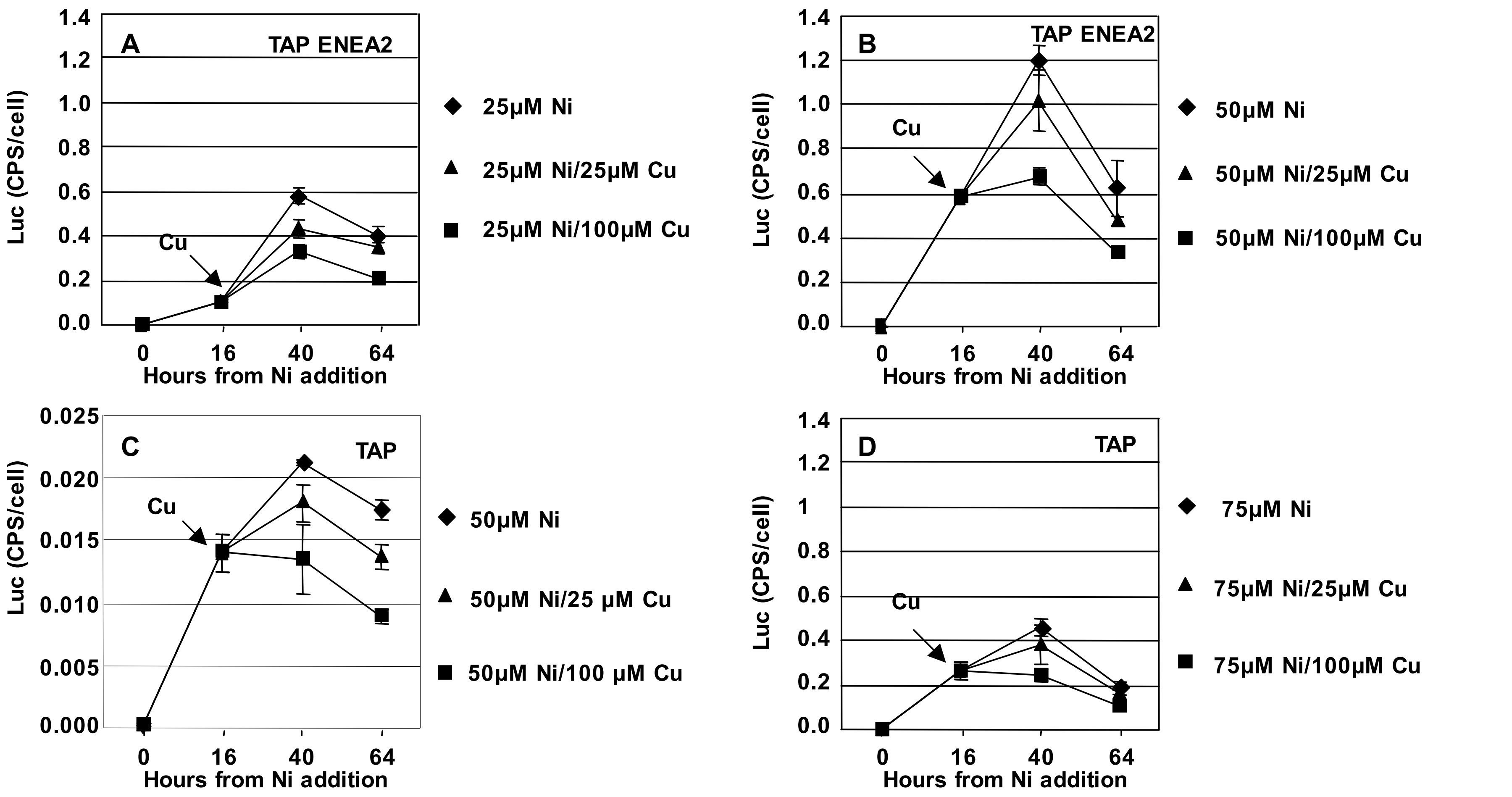

Figure S2: LUC activity in cultures induced with Ni in TAP ENEA2 medium (A and B), or TAP medium (C and D), and supplemented with different concentrations of Cu 16 hours after Ni addition.
