# Supplementary material for: An Optimized, Chemically Regulated Gene Expression System for Chlamydomonas
Source: PLoS One. 2008 Sep 12;3(9):e3200. doi: 10.1371/journal.pone.0003200 (PMC2527658; doi:10.1371/journal.pone.0003200)
Supplement: Figure S3 — (0.05 MB PDF) [file pone.0003200.s003.pdf]

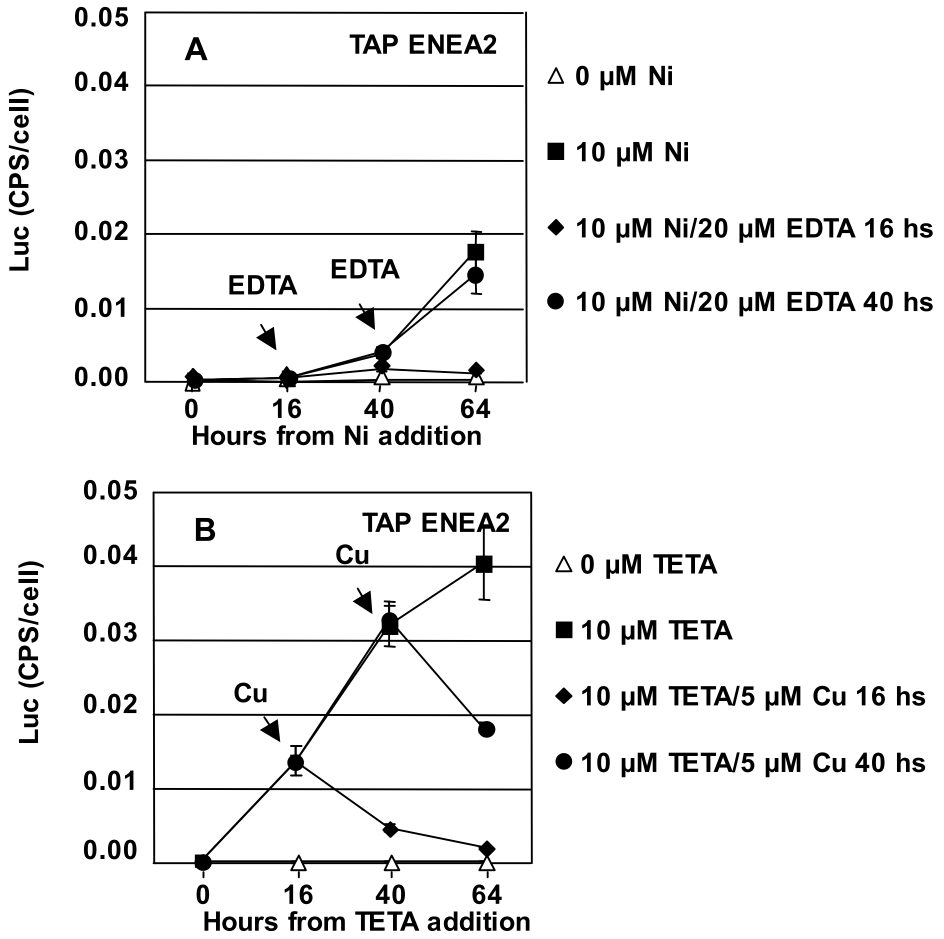

Figure S3: LUC activity in cultures induced with 10 µM Ni (A) or 10 µM TETA (B) in TAP ENEA2 medium and supplemented, respectively with EDTA and Cu at different times after induction.
