# Supplementary material for: An Optimized, Chemically Regulated Gene Expression System for Chlamydomonas
Source: PLoS One. 2008 Sep 12;3(9):e3200. doi: 10.1371/journal.pone.0003200 (PMC2527658; doi:10.1371/journal.pone.0003200)
Supplement: Figure S4 — (0.05 MB PDF) [file pone.0003200.s004.pdf]

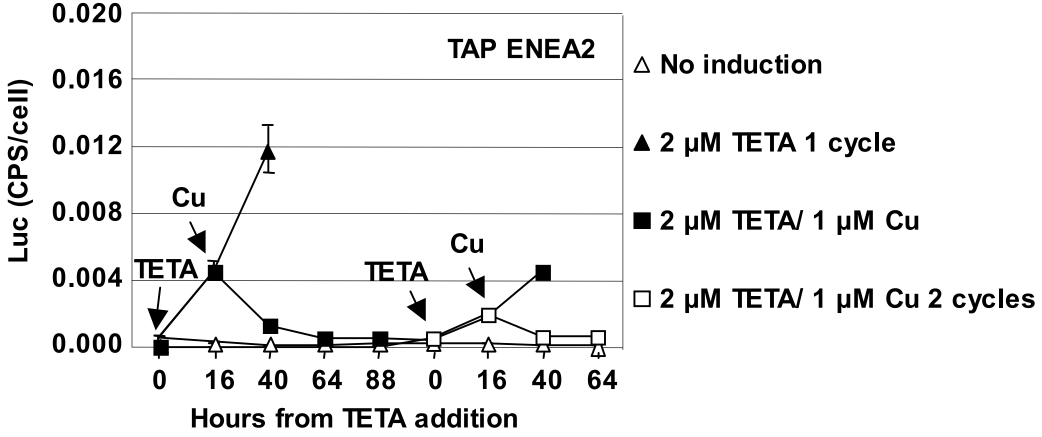

Figure S4: LUC activity in cultures induced with 2 µM TETA and repressed with 1 µM Cu for two subsequent cycles.
